# Curcumin as a Synergy Amplifier in Cancer Therapy

**DOI:** 10.3390/pharmaceutics18070825

**Published:** 2026-07-05

**Authors:** Sohail Mumtaz, Juie Nahushkumar Rana, Kainat Gul

**Affiliations:** 1 Department of Chemical and Biological Engineering, Gachon University, 1342 Seongnamdaero, Sujeong-gu, Seongnam-si 13120, Republic of Korea; 2 Fels Cancer Institute for Personalized Medicine, Lewis Katz School of Medicine at Temple University, Philadelphia, PA 19140, USA; 3 Department of Botany, Hazara University, Mansehra 21300, Pakistan

**Keywords:** curcumin, cancer combination therapy, chemosensitization, drug resistance, nanomedicine, co-delivery, nanostructured drug delivery systems, nanotechnology, pharmacokinetic synchronization, tumor microenvironment

## Abstract

**Background/Objectives:** Curcumin shows broad anticancer activity but limited clinical success as a standalone agent because of poor bioavailability and inconsistent tumor exposure. This review introduces the concept of curcumin as a molecular synergy amplifier and proposes that successful combinations depend on three interdependent determinants: mechanistic complementarity, suppression of adaptive resistance networks, and pharmacokinetic synchronization. **Methods:** Evidence on combinations with chemotherapeutics, natural bioactives, and nanotechnology-enabled delivery systems was critically evaluated, with emphasis on mechanism, resistance reversal, drug ratio, administration sequence, and tumor exposure. **Results:** Curcumin enhances therapeutic efficacy by sensitizing cancer cells, suppressing adaptive resistance pathways, targeting cancer stemness, and promoting multiple forms of programmed cell death. Importantly, analysis of current evidence indicates that therapeutic success depends not only on molecular synergy but also on pharmacokinetic synchronization between curcumin and partner agents. Many combinations demonstrating strong in vitro synergy fail to translate in vivo because optimal drug ratios, timing, and tumor exposure cannot be maintained. Nanotechnology-based co-delivery systems partially overcome these limitations through synchronized delivery and controlled release. **Conclusions:** Curcumin should be viewed as a molecular synergy amplifier whose clinical utility depends on mechanistic complementarity and pharmacokinetic synchronization with co-administered therapies. This framework provides a rationale for the design of next-generation curcumin-based combination therapies and identifies key priorities for clinical translation.

## 1. Introduction

**Figure 1:**
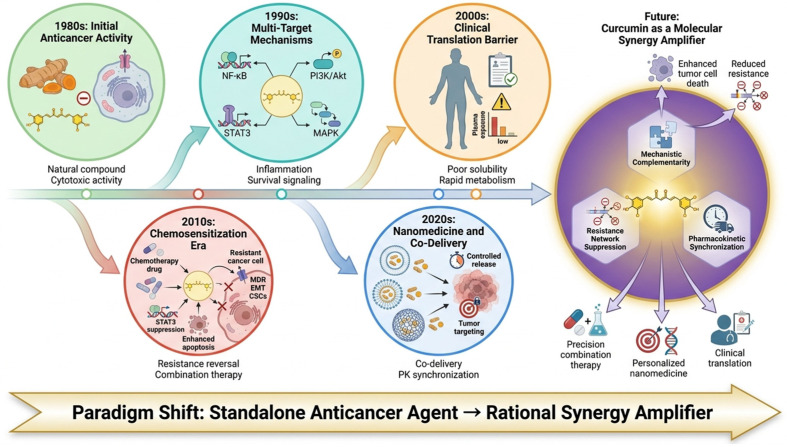
Evolution of curcumin in cancer therapy from a natural anticancer agent to a molecular synergy amplifier. The timeline presents representative decade-level milestones in the conceptual progression of curcumin research, from initial anticancer investigations in the 1980s to multi-target signaling studies, recognition of clinical translation barriers, chemosensitization strategies, and nanomedicine-enabled co-delivery systems.

Despite more than four decades of intensive research, curcumin has not achieved the widespread clinical adoption as an anticancer therapeutic as expected [1]. This apparent paradox arises because curcumin demonstrates potent anticancer activity across numerous experimental models while producing comparatively modest clinical outcomes [2]. Traditionally, this discrepancy has been attributed to poor bioavailability, rapid metabolism, and limited tumor accumulation [3,4,5]. However, emerging evidence suggests a more important explanation: Curcumin may not function optimally as a standalone therapeutic agent. Instead, its greatest value may lie in its ability to amplify the efficacy of other anticancer interventions [6,7,8,9]. Recent studies consistently demonstrate that curcumin enhances the activity of chemotherapeutic drugs, sensitizes resistant tumors, suppresses adaptive survival pathways, and improves the performance of advanced nanomedicine platforms. Nevertheless, not all curcumin-based combinations produce meaningful therapeutic benefit. While some combinations generate profound synergistic responses, others yield only additive effects or fail during translation from preclinical models to clinical settings. The mechanisms responsible for these divergent outcomes remain insufficiently understood. Over the past four decades, the scientific perception of curcumin in cancer therapy has progressively evolved from that of a natural anticancer compound with direct cytotoxic activity to that of a multifunctional adjunct capable of modulating oncogenic signaling, overcoming therapeutic resistance, and enhancing the efficacy of combination therapies. This conceptual evolution has culminated in the emerging view of curcumin as a molecular synergy amplifier that may facilitate the rational design of next-generation precision combination therapies (Figure 1).

The transition from viewing curcumin as a standalone cytotoxic agent to considering it a component of rational combination therapy is clinically important because many promising experimental effects have not been reproduced in patients. Direct anticancer activity is often demonstrated under exposure conditions that are difficult to maintain clinically, whereas rapid metabolism, variable bioavailability, formulation heterogeneity, and incomplete tumor delivery can reduce effective curcumin concentrations in vivo [2,3,4,5]. Moreover, the addition of curcumin to another treatment does not itself guarantee synergy. Inappropriate dose ratios, treatment sequence, or exposure duration may produce only additive effects, while persistent drug-efflux, survival, stemness, DNA-repair, or metabolic pathways may permit adaptive resistance despite initial tumor-cell injury [6,7,8,9,10]. The synergy-amplifier framework addresses these translational failures by requiring three forms of alignment: a partner therapy that is mechanistically complementary to curcumin, demonstrable suppression of adaptive resistance networks, and a formulation or dosing schedule that synchronizes both agents within the tumor at an effective ratio and for and effective duration. The framework therefore converts the conceptual evolution of curcumin research into practical criteria for prioritizing combinations that are more likely to retain efficacy beyond preclinical models.

Curcumin, a lipophilic polyphenol extracted from the turmeric root *Curcuma longa*, is one of the most widely investigated natural compounds for cancer therapy, owing to its pleiotropic properties, including anti-inflammatory, antioxidant, and anticancer properties [11,12]. Curcumin exerts its effects via the regulation of various signaling pathways involved in the development of cancer, including nuclear factor kappa B (NF-κB), phosphoinositide 3-kinase/protein kinase B (PI3K/PKB), signal transducer and activator of transcription 3 (STAT3), and mitogen-activated protein kinase (MAPK) pathways [13,14,15,16]. By targeting these pathways, curcumin is able to modulate the hallmarks of cancer, such as proliferation, apoptosis, angiogenesis, metastasis, and immune evasion [17]. Besides its impact on bulk tumor mass, recent studies have shown that curcumin can potentially regulate cancer stem cells (CSCs), regulate the components of the tumor microenvironment (TME), and regulate non-apoptotic cell death pathways, including ferroptosis and pyroptosis [18,19,20]. These mechanisms make curcumin an attractive multi-functional therapeutic that can overcome drug resistance and resolve cancer treatment failure. Despite these promising results, clinical outcomes with curcumin have been largely disappointing due to its poor water solubility, rapid degradation, low systemic exposure, limited systemic bioavailability, and poor target-site accumulation in vivo [3,21].

In an effort to overcome these challenges, combination therapies employing curcumin have emerged as a promising approach to improve therapeutic efficacy [22]. When combined with conventional chemotherapy drugs, curcumin exerts chemo-sensitizing effects, reverses multidrug resistance (MDR), inhibits pro-survival signalling pathways, and enhances cytotoxicity while reducing toxicity [15,23]. Likewise, combined use with other natural products, such as piperine and resveratrol, has also improved pharmacokinetic properties and demonstrated synergistic responses against cancer [24]. In addition, the strides made in nanotechnology have allowed curcumin-loaded nanoparticles, liposomes, and co-delivery of curcumin to be developed, enhancing solubility, stability, and delivery to tumors [25,26,27,28].

However, there are considerable translational uncertainties surrounding the potential of multi-modal curcumin-based therapies. The observed synergism often arises from in vitro experiments using supraphysiological concentrations that cannot be obtained clinically, making it doubtful whether this can be translated to the clinic [29]. Moreover, the lack of consistency in formulation approaches, the absence of a well-defined dose, and the lack of a biomarker-based selection of patients also hamper the clinical translatability [30]. Therefore, there is a pressing need for a holistic and mechanistic assessment of curcumin-based combinational strategies that link the experimental and clinical realms.

The ability of curcumin to act as a chemosensitizer, resistance modulator, and adjunct to anticancer therapies has been described previously [6,7,8,9,10,31,32,33,34]. Therefore, the contribution of the present review is not the first proposal that curcumin can enhance the activity of partner therapies. Rather, we organize the available evidence into an integrative framework comprising three interdependent determinants of therapeutically meaningful synergy: (i) mechanistic complementarity between curcumin and the partner therapy, (ii) suppression of adaptive resistance networks, and (iii) pharmacokinetic synchronization sufficient to maintain effective drug ratios and exposure at the tumor site. This framework is applied consistently across combinations with conventional anticancer drugs, natural bioactive compounds, and nanotechnology-enabled delivery systems. By linking molecular potentiation with formulation, delivery, drug exposure, quantitative outcomes, and clinical development, the review also explains why combinations displaying strong in vitro synergy may fail during in vivo or clinical translation.

### 1.1. Literature Search Strategy and Study Selection

This article was prepared as a structured critical narrative review. Relevant literature was identified through searches of PubMed/MEDLINE, Scopus, and the Web of Science Core Collection. Google Scholar and the reference lists of relevant original studies and review articles were additionally examined to identify eligible publications not retrieved through the primary database searches. Earlier landmark studies were retained when necessary to establish the historical or mechanistic context of curcumin research.

Search terms were used individually and in Boolean combinations, including “curcumin,” “curcuminoids,” “cancer,” “tumor,” “combination therapy,” “synergy,” “chemosensitization,” “drug resistance,” “cancer stem cells,” “ferroptosis,” “pyroptosis,” “phytochemicals,” “nanocarriers,” “nanoparticles,” “liposomes,” “co-delivery,” “controlled release,” “pharmacokinetics,” and “tumor targeting.” Searches were supplemented with the names of individual chemotherapeutic agents, natural bioactive compounds, delivery materials, and molecular pathways discussed in the manuscript.

#### Use of Generative Artificial Intelligence

During the preparation of this work, all literature searches, critical evaluation of studies, data synthesis, interpretation of findings, and initial drafting of the manuscript were performed manually by the authors. The fundamental concepts and scientific conclusions presented in this review were derived solely from the authors’ independent analysis of the published literature. ChatGPT (version GPT-5.5; OpenAI, San Francisco, CA, USA) was subsequently used solely to improve language quality, simplify sentence structure, and enhance the clarity and readability of the manuscript, including by assisting in the presentation of complex mechanistic concepts in a more accessible manner for readers. After using this tool, the authors reviewed and edited the content as needed and took full responsibility for the publication’s content.

## 2. Mechanistic Basis of Synergy in Curcumin-Based Combination Therapies

**Figure 2:**
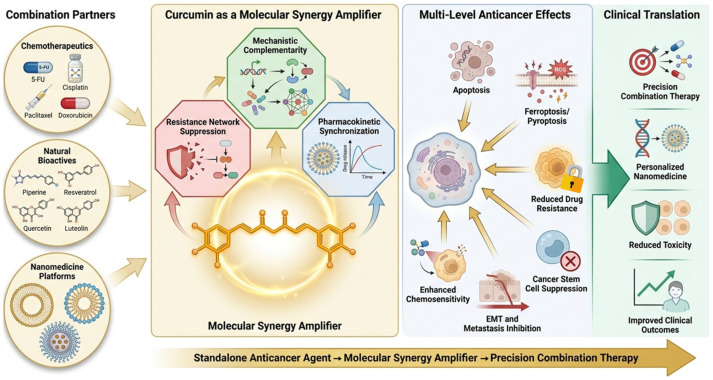
Conceptual Framework Governing Successful Curcumin-Based Combination Therapy. Curcumin acts as a multi-target synergy amplifier when combined with chemotherapeutics, phytochemicals, and nanodelivery systems to enhance anticancer efficacy. The figure also highlights major translational challenges and emerging future strategies aimed at improving the clinical success of curcumin-based therapies.

Curcumin-mediated synergy in cancer therapy arises from its unique ability to modulate multiple interconnected signaling networks while complementing the target-specific actions of conventional anticancer agents. Unlike single-target drugs, curcumin exerts pleiotropic effects that influence cellular redox balance, inflammatory signaling, and survival pathways, thereby creating a cellular context that enhances the efficacy of co-administered therapies [14,18,35,36]. This multi-target modulation is especially important in resistant tumors, where pathway redundancy and compensatory signaling frequently reduce the success of monotherapies. The overall conceptual framework linking synergistic combination strategies, translational challenges, and future development directions for curcumin-based cancer therapy is summarized in Figure 2.

### 2.1. The Three Pillars of Curcumin-Mediated Synergy

**Figure 3:**
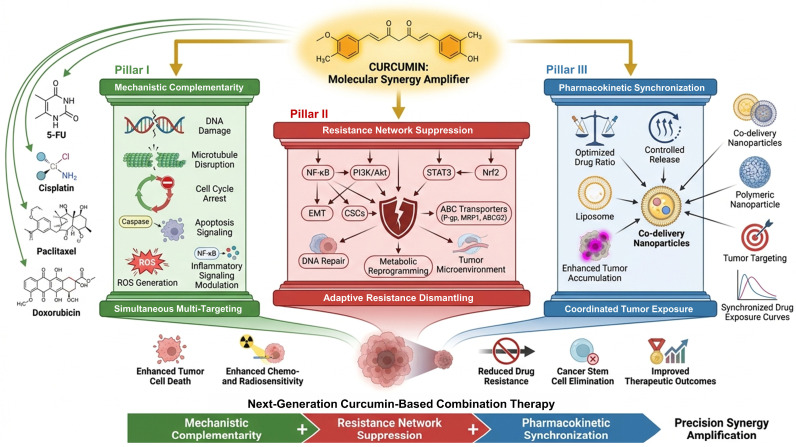
The three pillars governing curcumin-mediated therapeutic synergy in cancer combination therapy. Curcumin acts as a molecular synergy amplifier through mechanistic complementarity, resistance-network suppression, and pharmacokinetic synchronization. Together, these pillars enhance tumor-cell death and treatment sensitivity, reduce adaptive resistance and cancer stemness, and support more effective curcumin-based combination therapy.

Accumulating evidence indicates that the therapeutic value of curcumin extends beyond its direct anticancer activity and includes its capacity to enhance the efficacy of co-administered anticancer agents [6,7,8,9,10]. Based on the evidence synthesized in this review, curcumin-mediated synergy can be organized into three interconnected pillars (Figure 3). The first pillar, mechanistic complementarity, reflects curcumin’s ability to modulate multiple oncogenic, inflammatory, redox, and cell-survival pathways while complementing the target-specific actions of conventional therapies [10,14,18,35,36,37,38,39,40]. The second pillar, resistance-network suppression, includes inhibition of pro-survival signaling, multidrug-efflux transporters, cancer stemness, epithelial–mesenchymal transition, DNA-repair responses, and metabolic adaptations that sustain treatment resistance [41,42,43,44,45]. The third pillar, pharmacokinetic synchronization, concerns the maintenance of therapeutically effective drug ratios and coordinated tumor exposure through controlled release, nanocarrier-mediated co-delivery, and improved tumor accumulation [10,25,26,27,28,46]. Together, these mechanisms can enhance tumor-cell death and chemo- or radiosensitivity while reducing drug resistance, stem-like phenotypes, tumor progression, and metastatic potential [47,48,49,50,51]. This three-pillar framework, therefore, provides an evidence-based rationale for the development of mechanism-guided curcumin combination therapies.

### 2.2. Chemosensitization and Reversal of Drug Resistance

An important mechanism of curcumin synergy is its use as a chemosensitizer. Curcumin has been reported to inhibit major pathways responsible for drug resistance, such as the NF-κB, STAT3, and PI3K/Akt pathways, which are often highly active in resistant cancer cells [41,42]. This suppresses the expression of anti-apoptotic proteins (e.g., Bcl-2 and survivin) and activates pro-apoptotic proteins (e.g., Bax), restoring sensitivity to chemotherapy. Curcumin also has effects on adenosine triphosphate-binding cassette transporters such as P-glycoprotein, which mediate the efflux (pump) and retention of drugs within cells [43,52,53]. This phenomenon has been shown to lead to increased retention of drugs such as paclitaxel and doxorubicin with consequent improved cytotoxicity against resistant cancer cells. More recently, curcumin has been shown to inhibit epithelial–mesenchymal transition (EMT) and stem-like properties as well, both of which are involved in tumor resistance and relapse [54].

### 2.3. Multi-Target Complementarity and Network Modulation

Curcumin’s synergism with chemotherapeutic drugs is not simply the result of additive toxicity but also due to complementary effects on shared but discrete molecular targets. While many chemotherapy agents target specific aspects of cell physiology (e.g., DNA synthesis) or structure (e.g., microtubules), curcumin has wide-ranging effects on signaling pathways involved in regulating survival, inflammation, and oxidative stress [10]. This allows for simultaneous targeting of several cancer hallmarks, thus preventing the emergence of response-adaptive mechanisms. For example, curcumin-induced downregulation of inflammatory cytokines and transcription factors can downregulate survival factors that would protect cancer cells from the action of chemotherapeutic drugs. In addition, curcumin-mediated production of ROS might sensitize cancer cells to chemotherapeutically induced oxidative stress, thus enhancing cell death [37]. Redox-sensitive ion channels may also contribute to curcumin-responsive signaling. In neural tumor models, curcumin has been reported to inhibit TRPM2-associated oxidative-stress signaling and alter PARP1 and caspase activity, although the relevance of this mechanism to other tumor types requires further validation [55]. Crucially, emerging systems biology studies indicate that curcumin is a “network modulator” that affects multiple elements of oncogenic pathways, rather than a single target [38,39,40]. This may be an attribute essential to its ability to potentiate the action of a broad range of drugs in disparate cancers.

### 2.4. Induction of Non-Apoptotic Cell Death Pathways

Curcumin is also increasingly being implicated in the activation of non-apoptotic modes of cell death, such as ferroptosis and pyroptosis, which have been shown to be important in the treatment of cancer [56,57]. Such cell death pathways are especially important in cancer types resistant to apoptosis-inducing therapies. Curcumin-associated ferroptosis has been related to the induction of oxidative stress, lipid peroxidation, and reduction of glutathione-dependent antioxidants [58,59]. Combining curcumin with other agents that increase oxidative stress or decrease antioxidant capacity can result in exaggerated ferroptotic cell death, resulting in greater therapeutic benefit. Curcumin analogues may further broaden this response; for example, bisdemethylcurcumin has been investigated as a glutathione-depleting agent that simultaneously promotes ferroptosis and cuproptosis in hepatocellular carcinoma [60]. Likewise, curcumin has been reported to induce pyroptosis by activating inflammasome-associated proteins and cleaving gasdermin proteins by caspases [57]. Pyroptosis, an inflammatory type of cell death, may also mediate immunogenic effects within the tumor through its ability to promote an immune response, which could increase the efficacy of combinations with immunotherapy.

### 2.5. Modulation of Cancer Stemness and Cellular Plasticity

Cancer stem cells (CSCs) present a major challenge to successful treatment due to their role in tumour development, progression, and resistance. Recent studies have aimed to study curcumin’s ability to target CSC-related signaling networks such as Wnt/β-catenin, Hedgehog, and Notch, leading to reduced stemness and increased responsiveness to anticancer treatment [61]. This may help to prevent recurrences in combination therapy through the elimination of both bulk and resistant CSC populations. Additionally, curcumin has been shown to disrupt transcription factors involved in stemness and plasticity, leading to a decrease in tumor aggressiveness and a better response to treatment [44,45,62]. These studies suggest the need to consider cell heterogeneity and plasticity in curcumin-based combination approaches.

### 2.6. Critical Considerations: Synergy Versus Additive Effects

While a large array of synergistic effects has been reported, it is important to critically consider whether the observed responses represent pharmacological synergy or simply additive toxicity. A concern with many preclinical studies is the use of curcumin at high concentrations, which are not clinically attainable [63]. Additionally, differences in experimental conditions, such as cell lines used, scheduling, and ratio of combinations, impair the interpretation of synergy. Mathematical tools, like combination index and isobologram analysis, are crucial for defining and quantifying synergy [64]. Careful consideration of these parameters will help in the identification of clinically beneficial combinations.

## 3. Types of Curcumin-Based Combination Strategies in Cancer Therapy

Curcumin has been widely investigated using combination-based approaches to address its inherent pharmacokinetic problems and to improve its anticancer activity. These strategies can be broadly grouped into three types: (i) combination with conventional chemotherapeutic agents, (ii) co-administration with other natural bioactive compounds, and (iii) integration with nanotechnology-based delivery systems. Each of these strategies has its own merits, but their efficacy relies on the synergy of the mechanism of action, pharmacokinetic properties, and achievable clinical exposure. Collectively, Table 1, Table 2 and Table 3 summarize 44 representative combination systems: 15 involving conventional chemotherapeutic agents, 17 involving natural bioactive compounds, and 12 based on nanotechnology-enabled delivery.

### 3.1. Curcumin in Combination with Chemotherapeutic Agents

**Figure 4:**
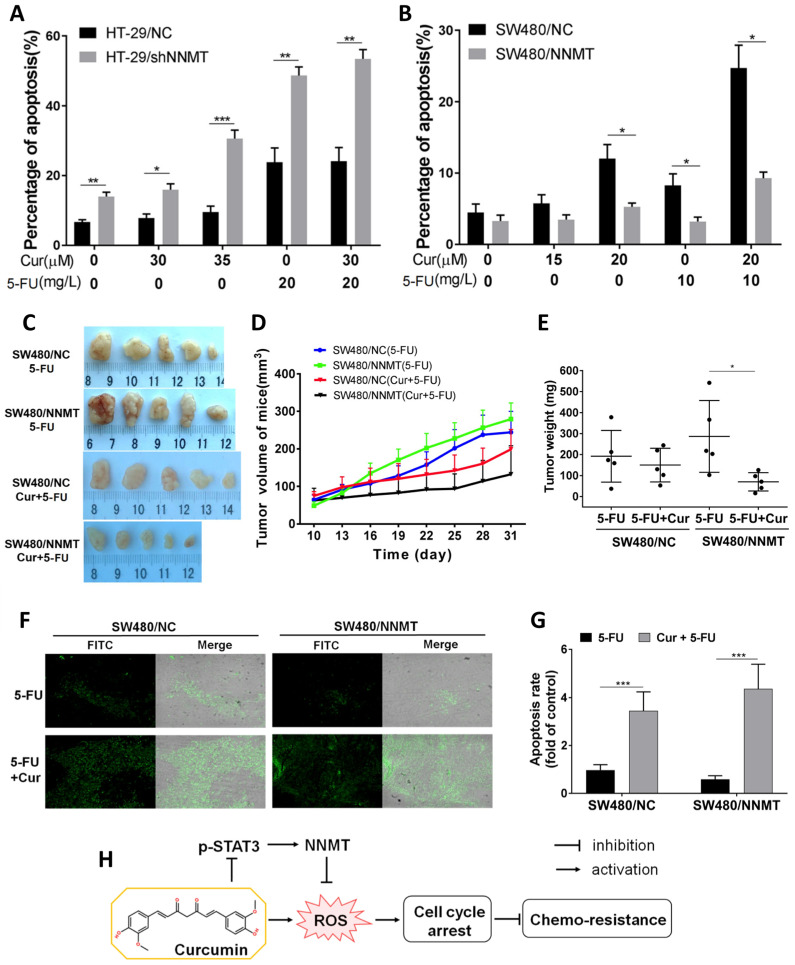
(**A**,**B**) Cell apoptosis with curcumin combined with 5-FU in HT-29 and SW480 cells. (**C**) Representative image showing tumors that were treated with 5-FU and/or Cur. (**D**) Tumor growth curves based on tumor volume from day 10 to day 31. (**E**) Tumor weights are displayed as a scatter plot across different groups. (**F**) Tumor sections analyzed for cell apoptosis using TUNEL staining (×100). (**G**) Apoptosis rates calculated from TUNEL staining and shown as a histogram, with the SW480/NC group treated with 5-FU serving as the control and assigned a value of 1. (**H**) Schematic depiction of how curcumin influences NNMT and chemosensitivity in CRC cells. Statistical significance is indicated as * *p* < 0.05, ** *p* < 0.01 and *** *p* < 0.001. Adapted from [50].

The use of curcumin in combination with conventional chemotherapies is perhaps the most thoroughly studied approach. Curcumin improves drug activity by mainly increasing chemosensitivity via inhibition of survival pathways, drug efflux pumps, and regulation of apoptotic proteins [47]. This results in increased drug uptake and desensitization of drug-resistant cancer cells. Curcumin has been shown to synergize with popular chemotherapeutic drugs, including 5-fluorouracil, cisplatin, doxorubicin, and paclitaxel. For example, curcumin increases the killing effect of 5-fluorouracil in colorectal cancer by blocking NF-κB-dependent mechanisms of resistance and triggering apoptosis [65,66]. Likewise, in cisplatin-resistant cancers, curcumin has been reported to suppress PI3K/Akt pathway activation and inhibit DNA repair mechanisms, thus sensitizing cells to the drug [67,68]. Beyond improving efficacy, curcumin could also mitigate systemic side effects of chemotherapy. Studies suggest that curcumin reduces oxidative stress and inflammation associated with chemotherapies and may improve the side effect profile of chemotherapy [69]. Although there is compelling preclinical data, there is limited clinical data on curcumin combination therapy, which needs to be evaluated under standardized conditions. A study reported that curcumin also reversed NNMT-driven 5-fluorouracil resistance by suppressing STAT3–NNMT signaling, increasing oxidative stress and apoptosis, and reducing tumor growth in vivo (Figure 4A–H) [50].

**Figure 5:**
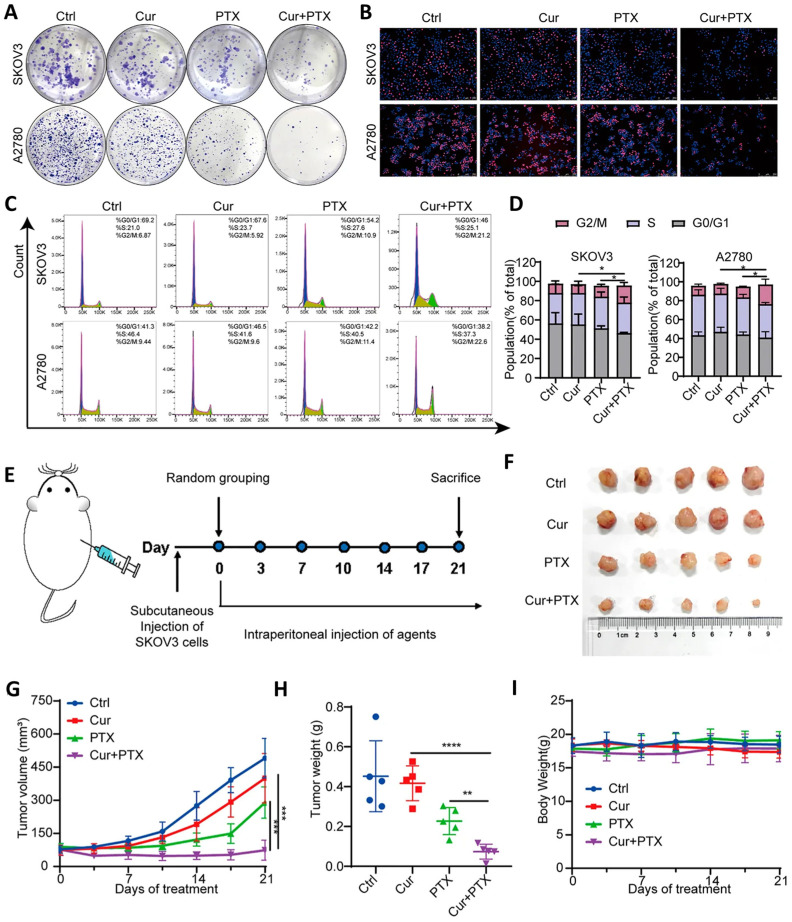
Curcumin increases the sensitivity of ovarian cancer cells to paclitaxel. (**A**) Clonogenic survival assay with decreased colony formation ability after treatment with curcumin (Cur), paclitaxel (PTX), and their combination (Cur + PTX). (**B**) Fluorescence staining showing a higher level of apoptosis in the Cur + PTX group. (**C**,**D**) Flow cytometry findings showing cell cycle distribution: The combination of Cur + PTX arrests the cell cycle in the G2/M phase and decreases the G0/G1 population in ovarian cancer cells. (**E**) Scheme of experimental design for in vivo experiments. (**F**) Photographs of tumors harvested from each group. (**G**) Tumor volume curves. (**H**) Tumor weight measurements revealed reduced weights in the Cur + PTX group. (**I**) Curves of tumor growth and body weight, showing inhibited tumor growth and lack of major side effects. Statistical significance is indicated as * *p* < 0.05, ** *p* < 0.01, *** *p* < 0.001, and **** *p* < 0.0001. Adapted from [70].

The combination effect of curcumin and paclitaxel was evaluated by Liu et al. [70] in ovarian cancer models, demonstrating a pronounced enhancement of anticancer activity compared to monotherapies. The colony formation assays performed by the authors revealed a substantial reduction in clonogenic survival following combined treatment (Cur + PTX), indicating a strong inhibitory effect on long-term proliferative capacity (Figure 5A). Similarly, fluorescence-based apoptosis analysis (Figure 5B) demonstrated a marked increase in apoptotic cell populations in the combination group, confirming enhanced cytotoxicity. Flow cytometric analysis further supported these findings, where the combination treatment significantly altered cell cycle distribution, promoting accumulation in the G2/M phase and reducing the G0/G1 population (Figure 5C,D), consistent with paclitaxel-mediated mitotic arrest potentiated by curcumin. The in vivo validation (Figure 5E–I) showed that Cur + PTX treatment led to a notable reduction in tumor volume and weight compared to control and single-agent groups, without significant changes in body weight, indicating effective tumor suppression with minimal systemic toxicity. Overall, these findings show that curcumin synergistically boosts paclitaxel-induced apoptosis and proliferation arrest both in vitro and in vivo, and also works to strengthen cell cycle arrest.

**Figure 6:**
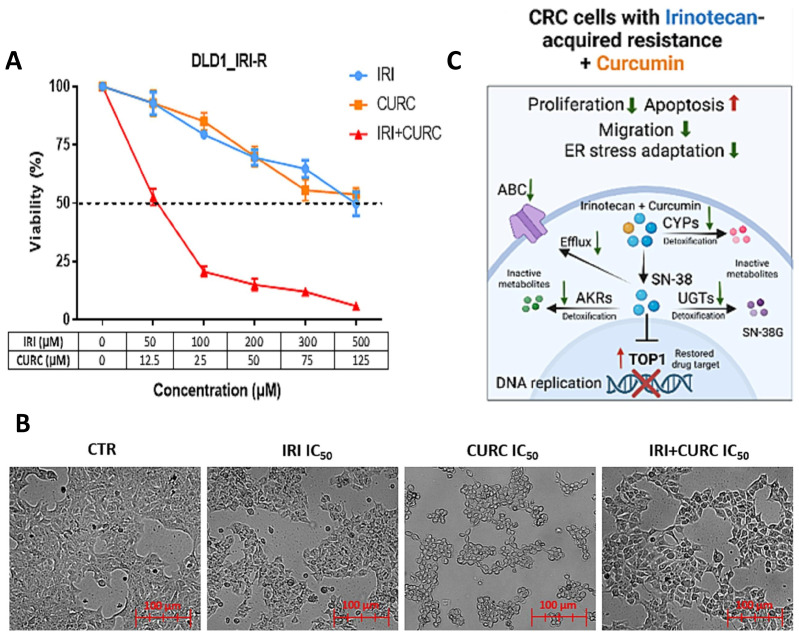
Curcumin-induced re-sensitization of IR-resistant colorectal cancer cells. (**A**) Cell viability assay of DLD1 irinotecan-resistant (DLD1_IRI-R) cells exposed to irinotecan (IRI), curcumin (CURC), or the combination (IRI + CURC), showing an increased killing effect and a synergistic effect with combination treatment. The dashed line indicates the 50% cell viability level. (**B**) Microscopic images of cell morphology changes in treated cells, with combination treatment leading to fewer and structurally changed cells compared to control and single-agent treatments. Scale bar: 100 μm. (**C**) Schematic of the proposed mechanism of curcumin-induced sensitization to irinotecan with increased induction of TOP1 and decreased expression of drug resistance proteins such as ABC transporters, CYPs, UGTs, and the ER stress pathway, resulting in increased apoptosis, decreased proliferation, and migration. Adapted from [71].

A recent report by Gavrilas et al. [71] offers strong evidence that curcumin successfully overcomes irinotecan resistance in colorectal cancer through multi-faceted molecular targeting. As shown in Figure 6, co-treatment (IRI + curcumin) led to a significant decrease in cell viability, with a notable drop below the 50% viability threshold (Figure 6A), suggesting a potent synergistic effect [71]. This was further supported by the morphological assessment, in which resistant cells treated with the combination showed significant loss of integrity and cell density compared to the control and monotherapies (Figure 6B). Molecularly, the interactive diagram (Figure 6C) illustrates that curcumin reverses resistance to irinotecan by upregulating TOP1 and simultaneously downregulating major resistance genes such as drug transporters (ABCs), metabolic enzymes (CYPs, UGTs), and ER stress response adaptation [71]. This broad disruption of targets not only boosts apoptosis but also suppresses cell proliferation and migration, underscoring curcumin’s potent chemosensitizing effects in bypassing acquired resistance by disrupting multiple network elements.

Curcumin-induced chemosensitization goes beyond conventional apoptosis and includes metabolic rewiring of chemo-resistant cancer cells. In particular, curcumin up-regulates miR-137, which directly inhibits glutaminase (GLS), leading to a metabolic blockade of glutamine metabolism, an important energy cycle in chemo-resistant colorectal cancer cells [53]. This process leads to resensitization to cisplatin, thereby establishing a new miRNA-metabolite axis of curcumin-based drug combinations [51]. Table 1 shows the synergy and major effects of curcumin with other drugs.

**Table 1:** Curcumin in combination with chemotherapeutic agents. (Arrows: “↑” indicates increase or activation; “↓” indicates decrease or inhibition).

Cancer/Model	Curcumin + Drug	Principal Mechanism	Key Outcomes	Ref.
Colorectal (SW620; in vitro/in vivo)	Curcumin + 5-FU	ERK/STAT1 signaling ↓; L1/Myc/Bcl-xL ↓; Apoptosis ↑	Chemosensitivity ↑; Tumor growth ↓; Survival ↑	[72]
Colorectal cancer (HT-29, SW480; in vitro/in vivo)	Curcumin + 5-FU	STAT3–NNMT ↓; ROS ↑; G2/M arrest ↑	5-FU sensitivity ↑; Tumor growth ↓; Apoptosis ↑	[50]
Ovarian (SKOV3, A2780; in vitro/in vivo)	Curcumin + Paclitaxel	miR-9-5p ↓; BRCA1/Bax ↑; G2/M arrest ↑	Colony formation ↓; Tumor growth ↓; CI < 1	[70]
Ovarian (paclitaxel-resistant; in vitro)	Curcumin + Paclitaxel	EGR1/SNIP1 ↑; NF-κB ↓; Bcl-2/Mcl-1 ↓	Paclitaxel resistance ↓; Apoptosis ↑	[48]
HNSCC (in vivo)	Curcumin + Docetaxel	MDSCs/M2 macrophages ↓; CD8^+^ T/NK cells ↑; IFN-γ/TNF-α ↑	Tumor burden ↓; Antitumor immunity ↑	[73]
Bladder (T24; in vitro and in vivo)	Curcumin + Cisplatin/Carboplatin + Gemcitabine	Aurora-A ↓; G2/M arrest ↑; Autophagy/Apoptosis ↑	Cytotoxicity ↑ (CI < 1); Tumor growth ↓	[74]
Ovarian (cisplatin-sensitive/resistant; in vitro)	Curcumin + Cisplatin (± DMC)	GSTP1/miR-133b ↓; Glutathione metabolism ↓; Apoptosis ↑	Cisplatin sensitivity ↑; Cell viability ↓	[75]
Breast cancer (MCF-7, MDA-MB-231; in vitro)	Curcumin + Paclitaxel	TGF-β ↓; PI3K/AKT/STAT3 ↓; COX2/NF-κB ↓	Paclitaxel resistance ↓; Migration ↓	[76]
TNBC (MDA-MB-231; in vitro)	Curcumin + Doxorubicin	ROS ↑; DNA damage signaling ↑; S-phase arrest ↑	Cell viability ↓; Apoptosis ↑; Normal-cell toxicity ↓	[77]
Ovarian (cisplatin-resistant; in vitro)	Curcumin (pre-treatment) + Cisplatin	TRXR1/mTOR/STAT3 ↓; Mitochondrial apoptosis ↑; ROS sensitivity ↑	Cisplatin sensitivity ↑; Cell viability ↓	[49]
Colorectal (irinotecan-resistant; in vitro)	Curcumin + Irinotecan	TOP1 ↑; ABC transporters/CYPs ↓; ER-stress signaling ↓	Irinotecan IC_50_ ↓ (~11-fold); Chemosensitivity ↑	[71]
NSCLC CSCs (in vitro)	Curcumin + Cisplatin	CSCs ↓; p21/Apaf1 ↑; Migration ↓	Chemosensitivity ↑; Proliferation ↓	[78]
NSCLC (in vitro/in vivo)	Curcumin + Cisplatin	Copper ↓; CTR1 ↑; Cisplatin uptake ↑	Apoptosis ↑; Tumor growth ↓	[79]
Colorectal (HT-29, LoVo)	Curcumin + Cisplatin	miR-137 ↑; GLS ↓; Glutamine metabolism ↓	Cisplatin sensitivity ↑; Proliferation ↓	[51]
Lungs (LL/2; in vitro and in vivo)	Curcumin + Doxorubicin (MPEG-PCL micelles)	Co-delivery ↑; Sustained release ↑; Angiogenesis ↓	Tumor growth ↓; Systemic toxicity ↓	[80]

**Figure 7:**
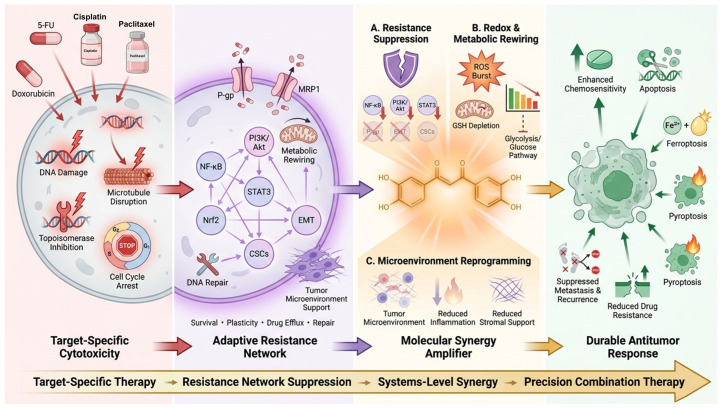
Mechanistic basis of curcumin-mediated synergy with pharmaceutical anticancer agents. Conventional anticancer drugs induce cytotoxic stress but may simultaneously activate survival, drug-efflux, repair, stemness, metabolic, and tumor-microenvironment pathways. Curcumin suppresses these adaptive responses, thereby increasing chemosensitivity, promoting programmed cell death, and reducing resistance, metastasis, and recurrence.

The synergistic interaction between curcumin and pharmaceutical anticancer agents arises from mechanistic complementarity at the systems level. Whereas conventional chemotherapeutics primarily target a single cellular vulnerability, such as DNA synthesis, microtubule dynamics, or topoisomerase activity, they simultaneously activate compensatory survival networks, including NF-κB, PI3K/Akt, STAT3, Nrf2 signaling, metabolic reprogramming, epithelial–mesenchymal transition, cancer stemness, DNA repair, and drug efflux mechanisms that ultimately promote resistance. In contrast, curcumin functions as a pleiotropic network modulator that concurrently suppresses these interconnected adaptive pathways, downregulates anti-apoptotic proteins and drug transporters, disrupts stemness signaling, and attenuates tumor microenvironment support. Moreover, by inducing ROS accumulation, mitochondrial dysfunction, and metabolic stress, curcumin lowers the threshold for apoptosis, ferroptosis, and pyroptosis while impairing the repair and survival mechanisms that enable tumor persistence (Figure 7). Consequently, synergy emerges not because curcumin adds another cytotoxic effect, but because it dismantles the biological networks that limit drug efficacy, thereby converting target-specific therapies into multi-level, systems-directed interventions with enhanced and durable anticancer activity.

### 3.2. Curcumin in Combination with Natural Bioactive Compounds

Natural–natural combinations are interesting in isolation from chemo-combinations because their value is not necessarily based on another chemical hit. In the best studies, the partner compound either provides additional pathway coverage, enhances uptake or retention, or changes the metabolic state from resistant to vulnerable [81]. This is why the best curcumin combinations are not always “naturals added together” but rather natural–natural combinations with a rationale: piperine as a bioenhancer and also as a stemness modulator, resveratrol and quercetin as multi-pathway polyphenols, epigallocatechin-3-gallate (EGCG) as an anti-angiogenic and modulatory catechin, and luteolin/apigenin as flavones to reinforce apoptosis and cell-cycle arrest signals [81]. The combinations yield superior responses in colon, breast, prostate, lung, melanoma, and bladder models. Some improve bio-uptake or retention, some thwart survival responses, some block EMT or stemness, others block tumor-associated metabolic or endothelial programs [82].

**Figure 8:**
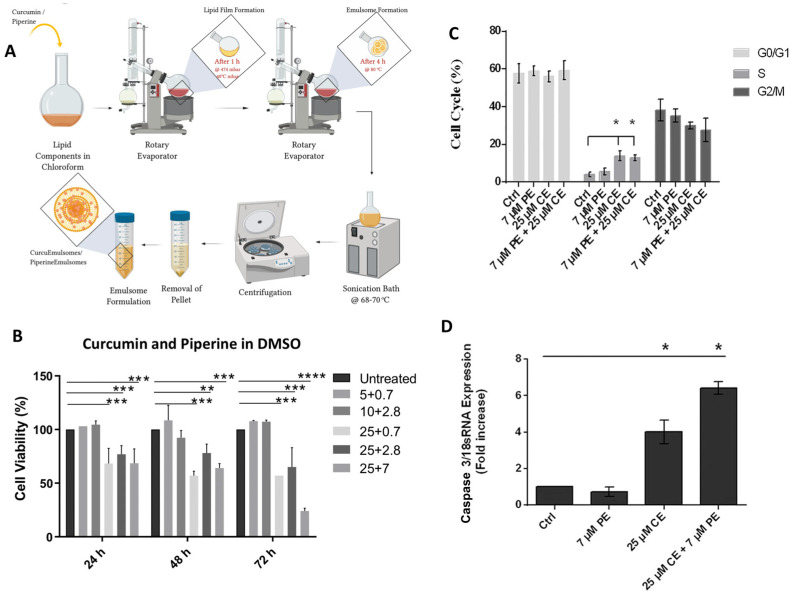
Improved anticancer efficacy of curcumin in nano-formulations in colorectal cancer. (**A**) Preparation of curcumin–piperine emulsomes. (**B**–**D**) Functional findings relating to enhanced cytotoxicity, cell cycle arrest, and apoptosis signaling with the combination treatment. Statistical significance is indicated as * *p* < 0.05, ** *p* < 0.01, *** *p* < 0.001, and **** *p* < 0.0001. Adapted from [82].

A recent paper by Bolat et al. [82] illustrates the significance of synergy with curcumin through facilitation of better bioavailability. Representatively illustrated in Figure 8A, nano-based co-delivery platforms (e.g., emulsomes) offer a means to address curcumin’s pharmacokinetic problems and enable effective cell loading. The efficacy of this approach is evident with increased cytotoxicity (Figure 8B) and exacerbated cell cycle arrest (Figure 8C), indicating that optimization of delivery is critical to curcumin efficacy, particularly in cancer therapy.

More importantly, the implications go beyond additive effects. The enhanced induction of apoptotic signals (Figure 8D) suggests that piperine is not a primary anticancer drug, but rather a biological enhancer that enhances curcumin dissolution, absorption, and cellular signaling. This has significant translational implications in that it reinforces the notion that curcumin-based interventions may need to be strategically combined with bio-enhancers or delivery partners to demonstrate clinical benefit. Thus, future studies must look towards pharmacokinetic/pharmacodynamic integration, with an emphasis on optimal design of carrier systems, timing, and ratios of drug combinations to achieve a high therapeutic window and minimal therapeutic variability. The pairing of curcumin and resveratrol represents a multi-targeted approach whereby the simultaneous inhibition of NF-κB and growth factor receptors (EGFR/IGF-1R) leads to greater tumor cell growth arrest and death [83]. Importantly, the effect in both p53 wild-type and p53 mutant models underlines the variable efficacy of this combination independent of genetic alterations in the tumor [83]. The combination of curcumin and piperine adds a crucial element to the phytochemical synergy by targeting the cancer stem cell population [81]. Through suppression of the Wnt pathway and subsequent targeting of ALDH-positive stem cells, this combination impairs self-renewal potential, an essential driver of tumor initiation, recurrence, and drug resistance [81]. These stemness-targeting approaches demonstrate the preventive and sustained therapeutic potential of curcumin combinations [81]. The delivery of curcumin and piperine in albumin-based nanoparticles exemplifies a desired synergy model with optimized pharmacokinetics, as therapeutic effectiveness is enhanced by increasing stability, cellular absorption, and overcoming multidrug resistance [84]. This shows that curcumin-based combinations may reach full anticancer potential through integration with designed delivery systems that compensate for poor bioavailability [84].

The combination of curcumin and quercetin is a representative example of multi-target, natural product synergy, in which co-inhibition of Wnt/β-catenin signaling and activation of the intrinsic apoptosis pathway collectively inhibit tumor growth [85]. Critically, the IC_50_ reduction reported by the authors in various cancer cell lines suggests that pharmacological synergy is being produced and that the combination of phytochemicals may increase efficacy by circumventing “pathway redundancy”—a common barrier to monotherapies [85]. In the case of curcumin and apigenin, the structural diversity of these molecules allows them to bind separate sites on tubulin, thereby allowing the dual targeting of microtubule dynamics [86]. This spatial distance (~19 Å) precludes competition for binding and allows cooperative disruption of cytoskeletal dynamics, leading to enhanced effects on mitotic arrest and apoptotic pathways [86]. This multi-site targeting strategy represents a promising approach to circumvent resistance to single-site microtubule-targeting agents [86,87]. Interactions between curcumin and genistein exemplify a dual-site endocrine-modulatory approach that cooperatively blocks estrogen receptor signaling and downstream kinase-dependent pathways [87]. Their complete suppression of proliferation evoked by a mixture of estrogenic pesticides suggests the promise of phytochemical integrations in preventing environmentally mediated carcinogenesis [87].

**Figure 9:**
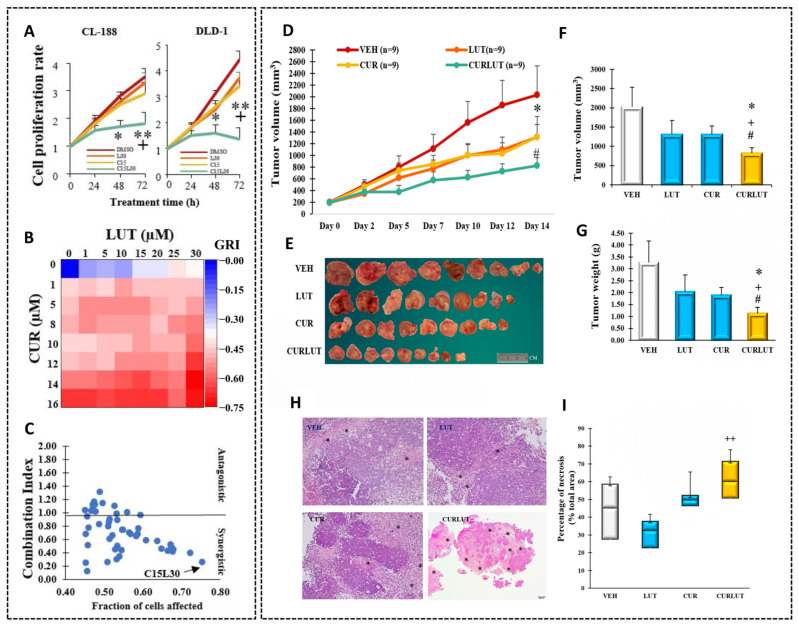
Synergistic anticancer effects of curcumin and luteolin in colorectal cancer. (**A**) Time profiling of single and combined treatments on cell proliferation in colon cancer cells. (**B**) Heatmap of combination screening for inhibition of growth using different drug combinations. (**C**) Combination index (CI) analysis showing synergistic effects at optimal dose ratios. (**D**) Tumor growth dynamics under vehicle, single-agent, and drug-combination treatment. (**E**) Images of resected tumors in various groups. (**F**) Tumor volume measurements. (**G**) Tumor weight. (**H**) Microscopic analysis (H&E stain) showing drug-induced changes and necrosis in the tumors. (**I**) Area of tumor necrosis. Statistical significance: in panel (**A**), * denotes comparison with the vehicle/DMSO control, whereas + denotes comparison of the combination with the corresponding single-agent treatments; * or +, *p* < 0.05; ** or ++, *p* < 0.01. In panels (**D**–**G**), *, +, and # denote comparisons with VEH, LUT, and CUR, respectively. In panel (**I**), ++ indicates *p* < 0.01 versus LUT. Adapted from [88].

The curcumin–luteolin combination is a dose-dependent phytochemical synergy model, tuned to discrete combination ratios (Figure 9A–C) [88]. This translates into distinct tumor growth suppression (Figure 9D–G), in line with the idea that curcumin needs co-effectors to remain active in vivo [88]. From a tissue-level perspective, the increased necrotic response (Figure 9H,I) further suggests that this interaction disrupts tumor architecture in addition to suppressing proliferation [88]. These observations support the growing notion that curcumin-based drug combinations work via multi-level, low-dose actions, presenting better translational opportunities than stand-alone therapies [88]. Additionally, another study demonstrated that the curcumin–luteolin combination is a next-generation model of phytochemical synergy that includes the activation of the immune response and inhibition of pro-tumorigenic processes [89]. In this study, the concurrent activation of interferon (IFN) signaling and suppression of transforming growth factor β (TGF-β)-mediated epithelial-to-mesenchymal transition (EMT) accentuates the interplay of this bidirectional regulatory axis, which inhibits both tumor growth and metastatic spread and resistance to treatment [89]. The effects on both sides of the spectrum highlight the capacity for phytochemical combinations to act as immune-modulatory strategies to fight cancer [89].

Shrestha et al. [90] reported a strong synergism between curcumin and melatonin in bladder cancer cells by suppressing the inflammatory and apoptosis pathways [90]. At the molecular level, melatonin enhances the effect of curcumin by blocking IKKβ kinase activity, converting NF-κB from a nuclear to a cytoplasmic location, thus repressing COX-2 gene transcription [90]. Simultaneously, the dual therapy induces mitochondrial apoptosis through increased cytochrome c release and caspase activation [90]. Crucially, the combined regulation of these two pathways leads to potent inhibition of cancer cell growth in vitro and in vivo, shedding new light on the promise of circadian regulation combined with phytochemicals [90]. Curcumin’s pairing with melatonin illustrates a two-action approach, as the drugs act in concert to silence pro-inflammatory NF-κB/COX-2 pathways and to activate mitochondrial apoptosis pathways, resulting in enhanced anticancer activity.

**Figure 10:**
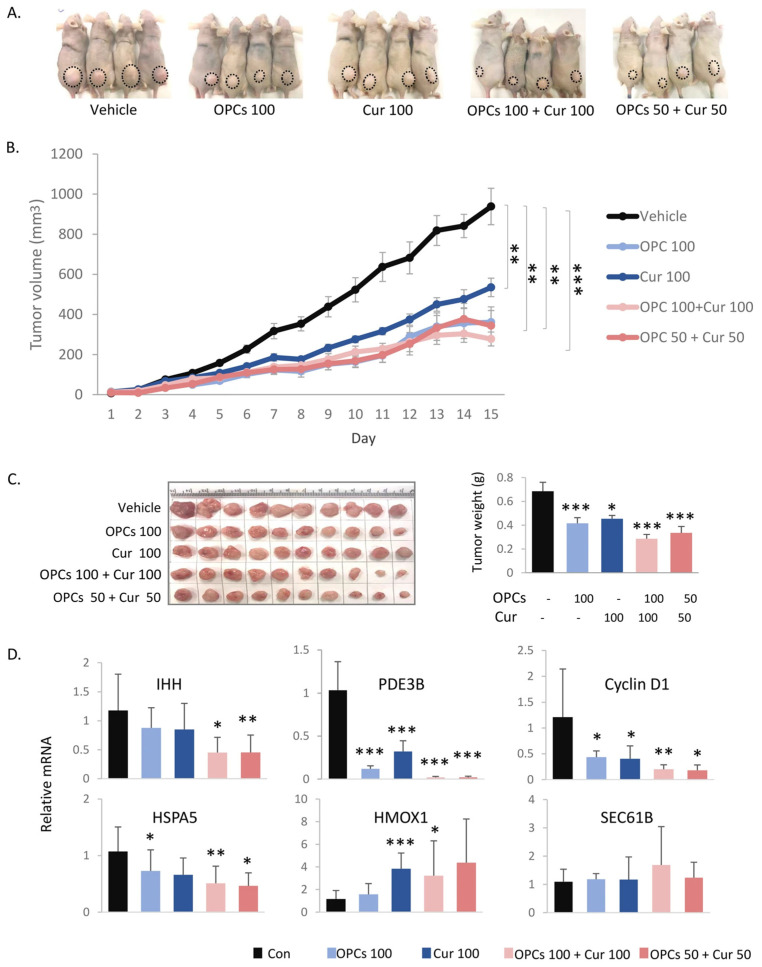
The in vivo antitumor efficacy and molecular modulation induced by the curcumin and OPC combination in colorectal cancer xenograft models. (**A**) Representative images of tumor-bearing mice following treatment with vehicle, OPCs, curcumin, and their combinations at different doses. (**B**) Tumor growth curves showing reduced tumor progression with combination treatment. (**C**) Representative images of excised tumors and corresponding tumor weight analysis. (**D**) Relative mRNA expression levels of key genes associated with proliferation, signaling, and stress response pathways. Statistical significance is indicated as * *p* < 0.05, ** *p* < 0.01 and *** *p* < 0.001. Reprinted from [91].

One of the best examples of pathway-level synergy between phytochemicals in colorectal cancers is found in the work of Ravindranathan et al. [91]. Rather than targeting a single, dominant pathway, curcumin and OPCs cooperatively down-regulate multiple cancer-promoting pathways, such as DNA replication, cell cycle, glutathione metabolism, protein export, and hedgehog signaling [91]. The in vivo significance of curcumin–OPC synergy is well supported by the findings of tumor suppression in xenografts [91]. As presented in Figure 10A–C, combination treatment results in a significantly enhanced suppression of tumor growth over single agents, and similar effects in tumors can be achieved at lower doses, thus demonstrating a dose-sparing synergistic effect [91]. This reinforces the concept that phytochemical combinations can enhance therapeutic efficiency while minimizing toxicity. At the molecular level, the cooperative nature of this interaction is reflected in the coordinated modulation of key oncogenic regulators (Figure 10D) [91]. The combination more effectively suppresses proliferation-associated genes (e.g., cyclin D1) and stress-adaptive markers (e.g., HSPA5), while concurrently altering metabolic and signaling mediators such as PDE3B and IHH. Such multi-target regulation supports a network-level inhibition of tumor progression, rather than reliance on a single pathway [91]. Collectively, these findings position curcumin–OPC combinations as a model of pathway-integrated synergy, where simultaneous disruption of proliferative, metabolic, and adaptive mechanisms enhances overall antitumor efficacy and reduces the likelihood of resistance [91].

**Figure 11:**
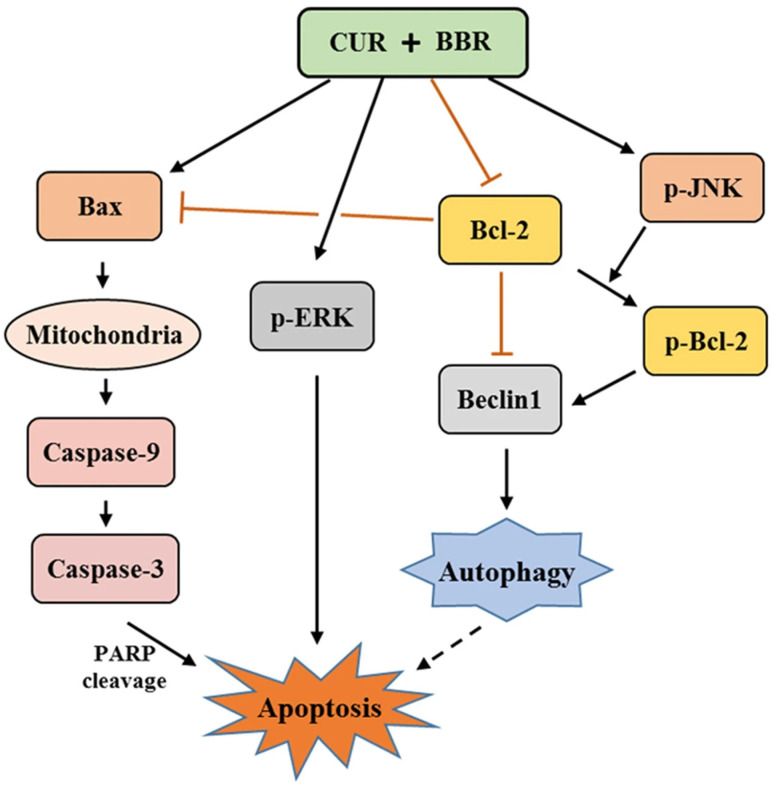
Suggested mechanism of synergistic cytotoxicity of curcumin (CUR) and berberine (BBR). The combination results in the activation of the ERK/JNK pathway, induction of mitochondrial (Bax) apoptosis (caspase-9/3, PARP cleavage), and enhanced autophagy regulation through Bcl-2/Beclin1 pathways, leading to increased cell death. Reprinted from [92].

Wang et al. [92] reported the combination effect of curcumin and berberine, which highlights a coordinated dual-cell-death strategy integrating apoptotic and autophagic signaling pathways. As illustrated in Figure 11, the synergy is mediated through activation of ERK and JNK pathways, which converge on mitochondrial apoptosis and autophagy regulation [92]. Increased expression of pro-apoptotic Bax and decreased expression of Bcl-2 alter their ratio and activate mitochondrial pathways, resulting in caspase activation and induction of apoptosis [92]. Similarly, JNK-induced changes in Bcl-2 phosphorylation prevent its association with Beclin1, thus initiating autophagy. This dual regulation allows the parallel induction of apoptosis and autophagy and suggests that synergism between curcumin and berberine occurs because resistance to cancer development is circumvented by multiple, interlinked apoptotic pathways.

Combinations of curcumin with other polyphenols are very sensitive to context. For instance, curcumin combined with (−)-epicatechin is synergistic in terms of antioxidant activity in polar (aqueous) systems but additive or even antagonistic in a lipid system, suggesting the importance of physicochemical properties in the effectiveness of combined systems [93]. These observations indicate that curcumin combinations are not necessarily synergistic but are influenced by the polarity, redox environment, and interaction state of the system, which may also have bearing on biological systems [93]. Table 2 shows the synergy and major effects of curcumin with other natural compounds.

**Table 2:** Curcumin in combination with natural compounds. (Arrows: “↑” indicates increase or activation; “↓” indicates decrease or inhibition).

Cancer/Model	Curcumin + Compound	Mechanism of Synergy	Key Outcomes	Ref.
Colorectal cancer	Curcumin + Piperine (Emulsomes)	Caspase-3 ↑; Apoptosis ↑; Cellular uptake ↑	Late apoptosis ↑ (~81%); Cell viability ↓ (~50%); Curcumin efficacy ↑	[82]
Colorectal cancer (in vitro/in vivo)	Curcumin + Resveratrol	NF-κB/EGFR/IGF-1R ↓; Apoptosis ↑; Proliferation ↓	Tumor growth ↓; Cell proliferation ↓; Effective in p53 wild-type and mutant cells	[83]
Multiple cancers (A549, HCT116, MCF7, A375; in vitro)	Curcumin + Quercetin	Wnt/β-catenin ↓; BCL2 ↓; Caspase-3/7 and PARP cleavage ↑	IC_50_ ↓; Apoptosis ↑; Proliferation ↓	[85]
Lung cancer (in vitro)	Curcumin + Apigenin	Dual tubulin targeting; G2/M arrest ↑; Bax/Bcl-2 ratio ↑	CI < 1; Apoptosis ↑; Microtubule disruption ↑	[86]
Breast cancer (MCF-7, in vitro)	Curcumin + Genistein	ER signaling ↓; Protein kinase activity ↓; Apoptosis ↑	Proliferation ↓; Growth inhibition ↑; Chemopreventive activity ↑	[87]
Colorectal cancer (in vitro/xenograft)	Curcumin + Luteolin	Notch1/TGF-β ↓; Proliferation ↓; Necrosis ↑	Tumor growth ↓; Migration/invasion ↓; Tumor necrosis ↑	[88]
TNBC (MDA-MB-231, BT-549; in vitro and xenograft)	Curcumin + Luteolin	IFN signaling ↑; TGF-β/EMT ↓; c-Myc/Notch1 ↓	Tumor growth ↓; Colony formation ↓; Immune response ↑	[89]
Breast cancer stem cells (in vitro)	Curcumin + Piperine	Wnt signaling ↓; ALDH^+^ CSCs ↓; Self-renewal ↓	Mammosphere formation ↓; Stemness ↓; No toxicity to differentiated cells	[81]
Breast cancer (MCF-7; in vitro)	Curcumin + Piperine (HSA nanoparticles)	Cellular uptake ↑; Bioavailability ↑; MDR ↓	Cell viability ↓; Drug stability ↑; Low toxicity	[84]
Bladder cancer	Curcumin + Melatonin	NF-κB/COX-2 ↓; Mitochondrial apoptosis ↑	IC_50_ ↓; Migration/invasion ↓; Tumor growth ↓	[90]
Colon cancer	Curcumin + Silymarin	Caspase-3/7 ↑; NF-κB ↓; Survival signaling ↓	Apoptosis ↑; Proliferation ↓; CI < 1	[94]
Colorectal cancer (including organoids)	Curcumin + oligomeric proanthocyanidins (OPCs)	DNA replication/Cell cycle ↓; Cyclin D1 ↓; Metabolic reprogramming	Proliferation ↓; Xenograft/organoid growth ↓; Dose reduction benefit	[91]
Breast cancer (MCF-7, MDA-MB-231)	Curcumin + Berberine	ERK/JNK ↑; Beclin1/LC3-II ↑; Bcl-2/p62 ↓	Apoptosis ↑; Autophagy ↑; Cell viability ↓	[92]
Colon cancer (HT-29, Caco-2)	Curcumin + Sulforaphane + Dihydrocaffeic acid	ROS ↑; ERK/JNK/p38 ↑; Mitochondrial apoptosis ↑	Cytotoxicity ↑ (CI < 1); Dose reduction; Selective cancer-cell toxicity	[95]
Liver cancer (HepG2)	Curcumin + Boswellic acids + Naringenin (PLGA NPs)	Cellular uptake ↑; Bioavailability ↑; ROS/Caspases ↑	IC_50_ ↓; Cytotoxicity ↑; Drug delivery efficiency ↑	[96]
Ehrlich ascites carcinoma (in vivo, mice)	Curcumin + Naringenin	VEGF/HIF-1α/HSP90/Akt ↓; Anti-angiogenesis ↑	Tumor burden ↓; Ascites ↓; Liver histology improved	[97]
NSCLC (in vitro/in vivo)	Curcumin + Baicalin (Nanoliposomes)	Bioavailability ↑; ROS ↑; Ki67 ↓; Apoptosis ↑	IC_50_ ↓ (CI = 0.5); Migration ↓; Tumor volume ↓; No toxicity	[98]

### 3.3. Curcumin in Nanotechnology-Based Combination Systems

**Figure 12:**
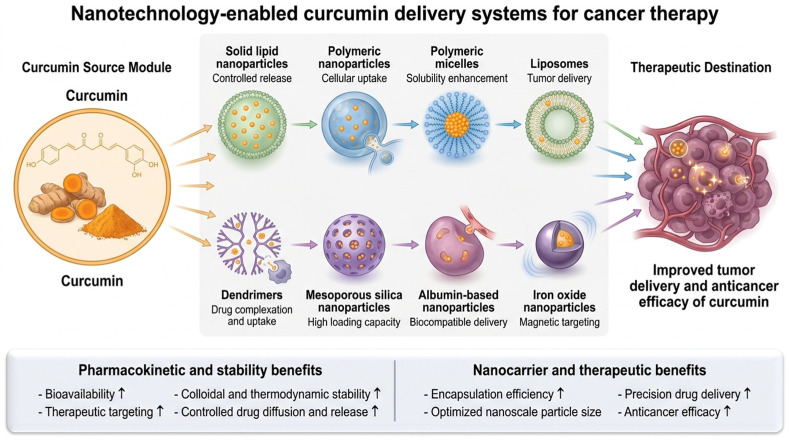
Nanotechnology-enabled curcumin delivery systems for cancer therapy. Major curcumin nanocarriers include solid lipid nanoparticles, polymeric nanoparticles and micelles, liposomes, dendrimers, mesoporous silica nanoparticles, albumin-based nanoparticles, and iron oxide nanoparticles. These platforms improve curcumin stability, solubility, encapsulation, controlled release, cellular uptake, tumor targeting, and overall anticancer efficacy.

The promising anticancer properties of curcumin are hindered by its low water solubility, metabolic lability, low bioavailability, and physicochemical instability. These pharmacokinetic challenges have long hindered its therapeutic use despite promising anticancer efficacy observed in vitro. These characteristics have been remedied through nanotechnology-based drug delivery systems, which are proving a game-changing approach, especially in combination with other drugs, to achieve synergistic anticancer properties (Figure 12). Nano-sized carriers, such as liposomes, polymer nanoparticles, micelles, nanoemulsions, and solid lipid nanoparticles, can achieve co-delivery of curcumin with other bioactive phytochemicals or chemotherapeutic drugs, which leads to improved cellular uptake, extended systemic circulation times, and sustained or controlled drug release [46,99]. Significantly, these systems are capable of enhancing not only pharmacokinetics but also pharmacodynamics, leading to superior therapeutic synergy over free-drug combinations.

Evidence is mounting that curcumin-based nano-combinations are more effective anticancer agents through a range of complementary actions [46,100,101]. These include (1) improved drug delivery and retention, (2) controlled drug release, (3) ROS modulation, (4) multi-targeting of signaling pathways, (5) suppression of tumor proliferation and metastasis, and (6) improved drug targeting and reduced side effects. Co-encapsulation in nanoliposomal formulations has shown strong synergies. For instance, combined curcumin–baicalin (mono-acetylated curcumin) nanoliposomes have significantly improved the therapeutic efficacy against non-small-cell lung cancer by promoting ROS generation and induction of apoptosis while preventing tumor proliferation and migration with highly synergistic effects (combination index, CI, <1). Crucially, nano-co-delivery systems enable spatiotemporal co-administration of drugs, enabling simultaneous delivery and action of both components within the tumor cell—a prerequisite for successful pharmacological synergy [99]. Additionally, the nano-delivery platforms can also circumvent resistance pathways, including efflux pumps and intracellular signaling pathways, to improve responsiveness to therapy. Overall, nanotechnology-mediated combination approaches comprise a new generation of therapeutic strategies, involving curcumin playing a dual role as a bio-active agent and a synergy enhancer in multi-drug delivery platforms.

In terms of the mechanistic improvement of curcumin, a number of studies also demonstrate the importance of the nanocarrier system. As an example, Arya et al. [102] demonstrate how the surface-engineered polymeric nanocarriers (PEG + chitosan) can boost the therapeutic potential of curcumin by overcoming pharmacokinetic and biological challenges. The significantly enhanced intracellular drug localization (NPs interact better with cells and show resistance to endolysosomal degradation) results in a stronger interaction with apoptotic-signal-regulating pathways. Remarkably, the suppression of metastatic properties (migration, invasion) shows that nanoformulations of curcumin are not only cytotoxic but also anti-metastatic. From a scientific viewpoint, the combination of functionalized systems such as hybrid PEG-chitosan represents a new-generation approach in which stealth (PEG) and bioadhesive (chitosan) properties are simultaneously achieved for enhanced circulation and targeting of cancer cells. Such a dual functionality is more important in aggressive cancers such as pancreatic cancer, where the challenge of drug delivery poses a significant barrier [102].

Likewise, co-encapsulation strategies also enhance curcumin’s therapeutic interventions. Co-encapsulation of curcumin with methotrexate within PLGA nanoparticles allows for co-delivery, triggering pro-apoptotic responses, limiting tumor growth, and preventing the drawbacks associated with mono-drug therapy [103]. Receptor-mediated (CD44 and FOLR1), targeted PEGylated PLGA nanoparticles significantly improve curcumin delivery, where targeted delivery to receptor-rich triple-negative breast cancer (TNBC) cells drives intracellular accumulation and therapeutic response [104].

Folic acid functionalization provides another active-targeting approach for tumors with elevated folate-receptor expression. Movileanu et al. reported that folic acid-decorated PEGylated magnetite nanoparticles enhanced the cellular uptake and anticancer activity of loaded curcumin in folate-receptor-expressing tumor cells [105]. In this system, PEG improved nanoparticle stability, folic acid enabled receptor-mediated recognition, and the magnetite core provided potential magnetic responsiveness. Although this formulation delivered curcumin alone rather than a therapeutic combination, it demonstrates a targeting strategy that could be adapted for synchronized co-delivery of curcumin and complementary anticancer agents.

The latest study marks a significant milestone in the field of curcumin-based nano-combination therapy, where curcumin is employed as a cytotoxic drug as well as a chemo-sensitizer to overcome MDR [106]. This effect is further enhanced by the presence of TPGS (a known irreversible inhibitor of P-glycoprotein), which inhibits drug efflux and thereby increases intracellular docetaxel accumulation [106]. Critically, the effectiveness of this nano-combination therapy in delivering injectable-like efficacy via the oral route represents a key breakthrough in clinical practice, circumventing both compliance and systemic toxicity problems. The enhanced pharmacokinetics, retention, and induction of reactive oxygen species (ROS) to trigger apoptosis indicate the benefits of using multifunctional nanocarriers to deal with therapeutic resistance [106]. Mixed micelles comprising TPGS and curcumin co-delivered with docetaxel successfully overcome MDR and allow an oral route of administration with similar efficacy to parenterally administered agents.

A key concept arising from such studies is that nanocarriers may not always lead to higher initial toxicity but instead prolong the therapeutic course. Curcumin systems involving nanocarriers may not be associated with an increase in initial cytotoxicity but rather with prolonged retention and durability, ultimately translating into sustained anticancer activity [107]. It is known that curcumin loaded into solid lipid nanoparticles (SLNs) experiences improved protection against immediate degradation and sustained release into cells, which prolongs the therapeutic duration and efficacy of curcumin despite lower initial cytotoxicity [108].

**Figure 13:**
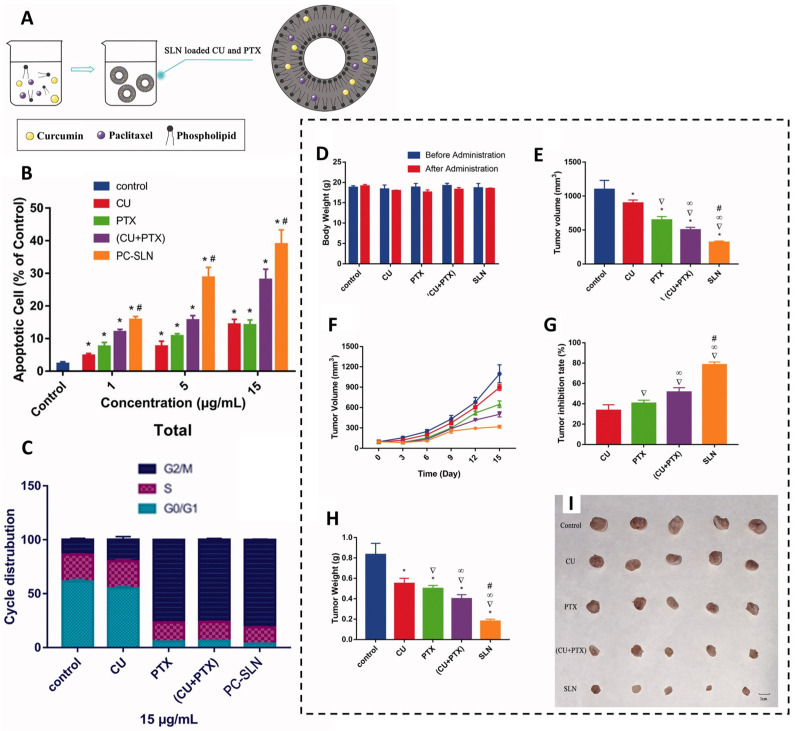
Co-delivery of curcumin and paclitaxel via solid lipid nanoparticles enhances anticancer efficacy. (**A**) Schematic image of the SLN co-loading system. (**B**) Apoptotic induction. (**C**) Cell cycle arrest. (**D**) Body weight analysis. (**E**) Tumor volume. (**F**) Tumor growth curve. (**G**) Tumor inhibition rate. (**H**) Tumor weight and (**I**) excised tumor pictures. Statistical significance was determined using the *t*-test: * *p* < 0.05 versus the control group; ▽ *p* < 0.05 versus the CU group; ∞ *p* < 0.05 versus the PTX group; and # *p* < 0.05 versus the CU + PTX group. Adapted from [109].

The clinical benefit of curcumin/paclitaxel combined therapy can be further amplified when the two drugs are co-encapsulated within a single nanocarrier. As shown in Figure 13A, solid lipid nanoparticle (SLN) formulation allows for co-encapsulation of curcumin and paclitaxel, leading to co-delivery and enhanced compatibility of the two drugs—a critical factor for sustaining optimal drug ratios during in vivo delivery, thereby achieving synergistic toxicity towards cancer cells [109]. At the functional level, this co-delivery approach translates to improved therapeutic outcomes at several levels of the target system. The nanoformulation further augments the intrinsic apoptosis response (Figure 13B) relative to free drugs, while also demonstrating a G2/M-phase arrest response (Figure 13C), indicative of a coordinated attack on cell division and survival. Critically, these are not simply additive effects but rather reflect enhanced intracellular drug concentrations and prolonged exposure afforded by the SLN system. The in vitro findings and system are clearly translated in vivo. The nano-co-delivery system is safe for systemic administration, with minimal body weight change (Figure 13D), and significantly retards tumor growth (Figure 13E,F) [109]. This improved efficacy is reflected by a higher tumor-inhibition rate (Figure 13G), reduced tumor weight, and smaller excised tumors (Figure 13H,I), confirming that precise co-delivery of curcumin and paclitaxel via nanocarriers indeed offers better anticancer efficacy than free drugs [109].

Carrier biocompatibility must be distinguished from the anticancer activity of the encapsulated or surface-conjugated therapeutic agent. Iron oxide nanoparticles provide magnetic responsiveness and may facilitate spatially controlled drug accumulation; however, their safety is influenced by particle size, surface charge, aggregation, coating stability, administered dose, intracellular degradation, and iron-mediated oxidative stress [110]. Surface modification with biodegradable polymers and polysaccharides can improve colloidal stability, reduce direct contact between the inorganic core and biological components, and provide functional groups for drug loading and tumor-targeting ligands [110].

Bourang et al. developed curcumin-loaded PLA–HA/Fe_3_O_4_ magnetic nanoparticles in which the iron oxide core provided magnetic responsiveness, while hyaluronic acid improved surface compatibility and offered potential CD44-mediated tumor targeting [111]. The formulation exhibited pH-dependent curcumin release and comparatively low intrinsic cytotoxicity of the unloaded carrier in HCT116 cells [111]. Nevertheless, because the biological evaluation was predominantly performed in a cancer-cell model, these findings cannot establish safety in nonmalignant cells or healthy tissues. A complementary system based on transferrin-functionalized PEG–PLGA magnetic nanoparticles was developed to enhance receptor-mediated and magnetically assisted curcumin delivery in lung cancer models [112].

More direct evidence of tumor selectivity was reported for curcumin-functionalized iron oxide nanoparticles administered with prolonged-release IFNα-loaded PLGA nanoparticles. The combined system produced enhanced cytotoxicity in A375 melanoma cells while exerting minimal effects on NIH-3T3 fibroblasts [113]. Polysaccharide modification can also regulate carrier stability, cellular interactions, and drug release. In a chitosan-modified, platelet-membrane-coated curcumin liposomal system, protonation of chitosan under mildly acidic conditions accelerated curcumin release within the tumor environment, whereas platelet-membrane proteins supported prolonged circulation and tumor-cell recognition [114]. This formulation showed favorable cytocompatibility and preliminary in vivo safety, with no evident abnormalities in body weight, major-organ histology, or serum biochemical indices [114]. These findings indicate that carrier composition, surface functionalization, receptor targeting, and stimulus-responsive release may widen the therapeutic window by increasing tumor exposure while limiting nonspecific toxicity. Nevertheless, a comprehensive evaluation should include matched nonmalignant cells, hemocompatibility, immune activation, biodistribution, carrier degradation, organ accumulation, and histopathological examination of healthy tissues [112].

Likewise, combined delivery of curcumin and tamoxifen with pH-responsive gemini surfactant nanoparticles has a profound effect on therapeutic responses by overcoming drug resistance, enhancing intracellular drug retention, and inducing synergistic apoptosis in breast cancer cells [115]. The design study by Santhamoorthy et al. [116] provides another insight into the function of the carrier surface and how it can regulate the loading and pH-sensitive release of curcumin, even though no additional therapeutic agent was co-loaded. The L-lysine groups simultaneously act as binding and release modulators that promote stronger drug retention under physiological conditions and rapid drug release in acidic media that mimic the more acidic target environments within tumors [116]. This approach supports the idea that functional-group engineering of mesoporous systems offers a promising strategy in curcumin nanomedicine, in particular for future combination systems. Some representative investigations and nano-combinations are listed in Table 3.

**Table 3:** Curcumin-based nanotechnology-enabled combination systems for enhanced anticancer therapy: mechanisms, delivery strategies, and therapeutic outcomes. Arrows indicate an increase or activation (↑) and a decrease or inhibition (↓).

Cancer/Model	Curcumin Nano-System + Co-Agent	Principal Mechanism	Key Outcomes	Ref.
Ovarian and breast cancer (A2780CP, MDA-MB-231; metastatic)	Curcumin-loaded PLGA nanoparticles (nano-CUR6)	Cellular uptake ↑; Sustained release ↑; Bcl-xL/Mcl-1 ↓; Caspase-9/PARP ↑	Uptake ↑ (2–6×); Clonogenicity ↓; Bioavailability ↑	[117]
Breast cancer (MCF-7, in vitro)	Curcumin-loaded PLGA nanoparticles	Stability ↑; Intracellular delivery ↑; Sustained release ↑; G2/M arrest ↑	Bioavailability ↑; Cell viability ↓; Safe nanocarrier	[118]
Pancreatic cancer (PANC-1, MiaPaCa-2, in vitro)	Curcumin-loaded chitosan/PEG-coated PLGA nanoparticles	Cellular uptake ↑; Bax/Caspase-3 ↑; Bcl-2 ↓; Migration/Invasion ↓	IC_50_ ↓ (2–3×); Cytotoxicity ↑; Intracellular accumulation ↑	[102]
Breast cancer (SK-Br-3, in vitro/in vivo)	Curcumin + Methotrexate co-loaded PLGA nanoparticles	Co-delivery ↑; Drug retention ↑; Sustained release ↑; Apoptosis ↑	IC_50_ ↓ (~2.5×); Tumor size ↓; Tumor incidence ↓	[103]
Triple-negative breast cancer (MDA-MB-231)	Curcumin-loaded PEGylated PLGA NPs (FA/HA/Tf ligands)	CD44/FOLR1/TfR1 targeting ↑; Cellular uptake ↑; Sustained release ↑	Cell viability ↓ (~8%); Targeted uptake ↑	[104]
Drug-resistant breast cancer (MCF-7/Adr, in vitro and in vivo)	Curcumin + Docetaxel mixed micelles (TPGS/Soluplus)	ROS ↑; P-gp ↓; Cellular uptake ↑; Oral absorption ↑	Bioavailability ↑ (~574%); Cytotoxicity ↑; Oral efficacy ≈ Taxotere	[106]
Breast cancer (in vitro/in vivo)	Curcumin-loaded solid lipid nanoparticles	Stability ↑; Sustained release ↑; Intracellular retention ↑	Bioavailability ↑ (1.25×); Cellular uptake ↑; Anticancer activity prolonged	[107]
Lung cancer (in vitro/in vivo)	Paclitaxel + Curcumin co-loaded SLNs	P-gp ↓; NF-κB ↓; Bax ↑/Bcl-2 ↓; G2/M arrest ↑	Tumor inhibition ↑ (78.4%); AUC ↑ (2.88×); IC_50_ ↓ (~7×)	[109]
Colorectal cancer (HCT116; in vitro)	Curcumin-loaded PLA-HA/Fe_3_O_4_ magnetic nanoparticles	CD44 targeting ↑; pH-responsive release ↑; Tumor targeting ↑	Cytotoxicity ↑; Acidic drug release ↑ (~98%); Normal-cell toxicity ↓	[111]
Breast (MCF-7), Osteosarcoma (MG-63)	Curcumin + GQDs-Fe_3_O_4_-FA	Folate targeting ↑; Magnetic guidance ↑; pH-responsive release ↑	Cell viability ↓; Sustained release ↑; Biocompatibility ↑	[119]
Breast cancer (MCF-7, MDA-MB-231)	Curcumin + Tamoxifen (TMX) in pH-responsive Gemini surfactant NPs	P-gp ↓; Bax/Bcl-2 modulation; ER inhibition ↑	CI = 0.561–0.353; IC_50_ ↓; Apoptosis ↑	[115]
TNBC (MDA-MB-231)	Curcumin-loaded L-lysine mesoporous silica nanoparticles (MS@Lys/Cur NPs)	Drug loading ↑; pH-responsive release ↑; Cellular uptake ↑	Curcumin loading ↑ (~68%); Cell killing ↑ (~90%); Hemolysis low (~3.5%)	[116]

The three combination strategies differ substantially in their strengths and translational readiness. Combinations with established anticancer drugs have the clearest therapeutic potential, but their benefits may be limited by systemic toxicity, resistance, and schedule-dependent antagonism. Combinations with natural bioactive compounds offer broad multi-target activity and potentially lower toxicity, yet their development is constrained by variable composition, overlapping mechanisms, poor bioavailability, and limited clinical validation. Nanocarriers are not therapeutic partners in themselves but enabling platforms that can improve curcumin stability, tumor accumulation, drug ratio control, and synchronized release; however, their translation requires rigorous safety, manufacturing, and regulatory assessment. Accordingly, no strategy is universally superior: conventional drug combinations currently provide the strongest clinical foundation, natural compound combinations remain largely exploratory, and nanotechnology offers the greatest potential to improve pharmacokinetic coordination across both categories.

### 3.4. Clinical Status of Curcumin-Based Anticancer Regimens

Clinical translation of curcumin-based anticancer strategies remains largely confined to early-phase studies. In metastatic colorectal cancer, oral curcumin was evaluated with FOLFOX chemotherapy in a Phase I/IIa program, including a randomized Phase IIa study showing that the combination was feasible and generally well tolerated [120]. A randomized, double-blind Phase II trial subsequently investigated intravenous curcumin in combination with paclitaxel in patients with advanced or metastatic breast cancer, providing preliminary evidence of clinical activity without a major increase in treatment-related toxicity [121]. Nevertheless, these studies were not sufficiently large to establish curcumin as a standard adjunct to chemotherapy.

Phase I investigations have focused mainly on safety, dose selection, and pharmacokinetic interactions. A Phase I dose-escalation study of an oral curcumin phosphatidylcholine complex combined with irinotecan in patients with advanced solid tumors found that the regimen was generally tolerable and did not produce a clinically meaningful alteration in irinotecan pharmacokinetics [122]. Intravenous liposomal curcumin has also undergone Phase Ib evaluation in patients with locally advanced or metastatic cancer. This study defined dose-related tolerability limitations, including hematological toxicity at higher doses, but did not demonstrate objective tumor responses according to RECIST criteria [123]. More recently, a registered Phase Ib/IIa study has evaluated liposomal curcumin with radiotherapy and temozolomide in patients with newly diagnosed high-grade gliomas, with emphasis on dose determination, safety, feasibility, and preliminary efficacy (NCT05768919) [124]. Larger randomized trials incorporating formulation-specific pharmacokinetics, clinically achievable exposure, biomarker-guided patient selection, and validated efficacy endpoints are required before curcumin-containing combinations can be incorporated into routine oncology practice.

### 3.5. Controversies and Contradictory Evidence

Despite substantial evidence of curcumin-mediated chemosensitization, beneficial interactions have not been observed consistently. For example, in breast cancer models, curcumin inhibited apoptosis induced by camptothecin, mechlorethamine, and doxorubicin by up to 70% and reduced cyclophosphamide-mediated tumor regression in vivo [125]. These findings contradict studies summarized in Table 1, in which curcumin enhanced doxorubicin activity. Such differences may reflect variations in cancer model, curcumin concentration, redox state, treatment sequence, and dependence of the partner drug on oxidative injury.

Clinical studies have also produced limited or inconsistent outcomes. In advanced pancreatic cancer, oral curcumin monotherapy produced prolonged disease stabilization in one patient and a brief tumor regression in another, but most participants did not experience meaningful clinical benefit, and systemic curcumin exposure remained low [126]. Intravenous liposomal curcumin improved systemic delivery but produced no objective tumor responses in a Phase I study and caused hemolysis or clinically relevant decreases in hemoglobin in some patients at the highest dose [123]. In metastatic castration-resistant prostate cancer, the addition of curcumin to docetaxel did not improve progression-free survival, overall survival, prostate-specific antigen response, or quality of life, and the study was stopped for futility [127]. Curcumin–gemcitabine studies have likewise yielded differing conclusions. One study in advanced pancreatic cancer reported gastrointestinal intolerance in several patients, limited antitumor activity, and poor feasibility of administering 8 g/day curcumin with gemcitabine [128]. Another Phase I/II study in gemcitabine-resistant pancreatic cancer considered the same curcumin dose feasible, although no complete or partial responses were observed and only a limited number of patients achieved stable disease [129]. Similarly, the randomized CUFOX study established that curcumin could be administered with FOLFOX, but its small sample size did not permit definitive conclusions regarding survival benefit [120].

These negative and contradictory findings do not eliminate the potential value of curcumin but establish important limits. Curcumin should therefore be regarded as a conditional modulator whose benefit must be demonstrated for each therapeutic partner. Collectively, these findings indicate that curcumin-mediated synergy cannot be assumed from simple co-administration. Successful combinations require alignment of the three proposed pillars: mechanistic complementarity, suppression of relevant resistance networks, and pharmacokinetic synchronization. Failure to satisfy one or more of these requirements may result in weak, null, or antagonistic effects despite promising preclinical rationale.

## 4. Integrated Perspective on Curcumin-Based Combination Strategies

Curcumin’s advancement from a multifunctional chemical to a systems-level modulator in combination therapies [10,130] hinges on its ability to alter the signaling environment and drug sensitivity, rather than its intrinsic chemotherapeutic activity. Across a broad range of cancers, curcumin consistently potentiates responses to conventional chemotherapeutics, natural bioactives, or nanoscale drug delivery technologies [9,131,132,133], via concerted inhibition of NF-κB, STAT3, PI3K/Akt, and MAPK signaling pathways and modulation of pro-apoptotic regulators such as Bax/Bcl-2 and caspases. Through this multimodal intervention, curcumin serves as a chemosensitizer, re-sensitizing resistant cancers to chemotherapeutic agents [134,135].

In conventional multi-drug chemotherapy regimens, curcumin improves efficacy largely by suppressing survival pathways and by reversing MDR [136]. Through demobilization of efflux pumps (e.g., P-glycoprotein) and transcriptional signatures of proliferation and inflammation, curcumin enables greater drug retention and activation of apoptosis [132]. Yet, while preclinical studies consistently show curcumin’s interactions, these are subject to narrow therapeutic windows, depending on tumor type, drug combinations, and temporal exposure. The lack of consistent synergy validation (e.g., combination index calculations) also obscures the data, pointing to a disconnect between mechanistic promise and therapeutic efficacy.

Interactions with natural bioactive agents further build on this concept by exploiting non-redundant signaling pathways [137,138,139]. When combined with molecules such as resveratrol, luteolin, or piperine, curcumin allows for concerted targeting of inflammatory, stemness, and proliferation-associated pathways such as the NF-κB, EGFR, IGF-1R, Notch, and TGF-β pathways [83,89,140,141,142]. Such combinations have the benefits of lower toxicity and coverage of a broader spectrum of signaling pathways, but are limited by pharmacodynamic mismatch. The common vulnerabilities of low solubility and high metabolism restrict circulation, and many in vitro bioactivity combinations are unlikely to translate into in vivo synergies. Consequently, the efficacy of phytochemical combinations tends to be overestimated in reductionist models, which neglect to account for absorption, distribution, and metabolism parameters.

Nanotechnology formulations have provided a major remedy to these challenges by converting curcumin-based synergists into pharmacokinetically aligned systems. Encapsulation into liposomes, polymeric nanoparticles, micelles, or solid lipids provides parallel release of drugs, adjustable release profiles, and improved tumoral retention, thereby ensuring that pharmacodynamics are congruent with pharmacokinetic systems [106,143,144,145,146,147]. Here, nanocarriers do not simply act as carriers but as facilitators of synergism, delivering multiple agents to the site of action in the right ratios and time frames. Additionally, stimulus-responsive (e.g., pH-responsive) systems and targeting ligands (e.g., CD44 or folate receptors) increase spatial selectivity, thereby reducing side effects and enhancing site-specific drug action.

### 4.1. Limitations of Current Evidence and Translational Challenges

Despite substantial progress, several limitations of the current evidence must be resolved before curcumin-based combinations can be translated into reliable clinical strategies. First, much of the available evidence originates from in vitro and animal studies that differ considerably in cancer models, curcumin formulations, doses, drug ratios, treatment sequences, and methods used to define synergy. These differences complicate direct comparison and may partly explain why apparently promising combinations produce inconsistent outcomes across experimental systems. Second, EPR-dependent tumor targeting is highly variable, limiting the predictability of nanoparticle accumulation. Third, several studies employ curcumin concentrations that are difficult to reproduce clinically and do not determine whether the proposed synergistic ratio is maintained in plasma or tumor tissue. Fourth, the biocompatibility of the complete nanoformulation, rather than only that of free curcumin, must be established in nonmalignant cells and clinically relevant in vivo models through assessment of hemocompatibility, immune responses, carrier degradation, biodistribution, organ accumulation, and histopathological injury in healthy tissues. Fifth, nanoparticle accumulation through the enhanced permeability and retention effect varies considerably among tumors and patients, limiting the predictability of passive targeting. Sixth, increasingly complex co-delivery systems introduce challenges related to formulation reproducibility, long-term stability, large-scale manufacturing, regulatory assessment, and preservation of the intended drug ratio during storage and administration. Finally, biomarker-guided patient selection and well-controlled clinical studies remain limited. Therefore, the three-pillar framework presented in this review should be regarded as a mechanism-based foundation for prioritizing and prospectively validating the most promising combinations rather than as an already established clinical treatment algorithm.

### 4.2. Future Perspectives

The key to advancing active curcumin therapy in cancer treatment is a shift from empirical combination approaches to mechanism-based rational design (i). Rather than using simple drug combinations, future studies should look at co-delivery systems via intelligent co-vehicles to ensure co-delivery, optimal drug ratios, and targeted delivery of compounds, especially via ligand- and stimulus-responsive nanoparticles (ii). In clinical practice, pharmacokinetic synchronization could be achieved through fixed-ratio co-delivery, formulation-guided scheduling, and pharmacokinetic–pharmacodynamic optimization. Co-delivery systems can maintain predefined drug ratios and coordinated release, as shown preclinically for curcumin combined with methotrexate, docetaxel, or paclitaxel [103,106,109]. When the agents are administered separately, human pharmacokinetic data, serial sampling, pharmacodynamic biomarkers, and population modelling can guide dose, sequence, and timing [122,123]. Synchronization does not require identical plasma profiles but rather sufficient overlap of biologically active exposure during the therapeutic window. Prospective studies are still required to confirm that coordinated systemic exposure results in coordinated tumor exposure.

**Figure 14:**
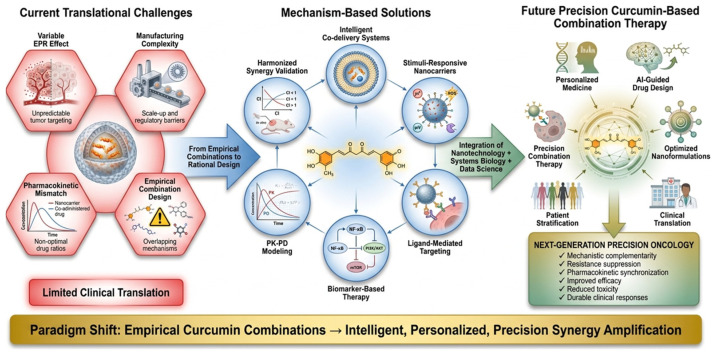
Roadmap toward precision curcumin-based combination therapy: challenges and future directions. Current limitations, including heterogeneous tumor targeting, manufacturing complexity, pharmacokinetic mismatch, and empirical combination design, are addressed through mechanism-based solutions integrating nanotechnology, biomarker-guided therapy, PK–PD modeling, and systems biology. These advances support the development of personalized, precision curcumin-based combination therapies with improved efficacy and clinical translation.

Significant improvements need to be made by combining this approach with biomarker-based therapy, where combinations are used in a population of patients selected by molecular signatures such as NF-κB, PI3K/Akt, or MDR pathways (iii). This naturally leads to personalized medicine, in which therapies are designed based on individual genetic, mutational, and resistance patterns, as well as personal pharmacokinetics (iv). Furthermore, the use of artificial intelligence (AI) and computational approaches is anticipated to revolutionize the research field by modeling the best drug combinations, identifying new targets for combination therapies, and designing effective nanoformulations with better delivery and lower toxicity (v). Crucially, future research should also focus on clinically relevant validation techniques, such as harmonized synergy methodology, PK–PD modeling, and robust in vivo and clinical studies to validate laboratory discoveries in the clinic (vi). The next chapter of curcumin research will be defined by integration, bringing together nanotechnology, systems biology, and data-based design, integrating curcumin as part of next-generation, precision oncology approaches (vii). Collectively, overcoming the current translational barriers of curcumin-based combination therapies will require a transition from empirical drug combinations toward mechanism-driven, biomarker-guided, and nanotechnology-enabled precision strategies that integrate systems biology and data-driven approaches (Figure 14).

## 5. Conclusions

The available evidence indicates that curcumin is most promising not as a standalone anticancer agent but as a molecular synergy amplifier capable of improving the activity of partner therapies by weakening cancer cell survival, therapeutic resistance, stemness, and adaptive signaling networks. However, mechanistic complementarity alone is insufficient to produce clinically meaningful synergy, because both curcumin and its partner agent must reach the tumor at therapeutically effective concentrations, ratios, and times. Pharmacokinetic synchronization is therefore fundamental to the successful translation of curcumin-based combinations. In this context, nanotechnology represents a particularly important enabling strategy, as co-delivery systems can improve curcumin stability and bioavailability, coordinate drug release, preserve effective drug ratios, and enhance tumor accumulation. Combinations with natural bioactive compounds may also provide broad multi-pathway activity and reduce treatment-related toxicity, although their clinical reliability remains constrained by variable composition, poor bioavailability, and inconsistent pharmacokinetic behavior. Consequently, successful combination therapy should prioritize the rational selection of mechanistically complementary agents that target distinct tumor vulnerabilities and suppress adaptive resistance rather than simply increasing the number of therapeutic components. Current clinical evidence remains limited to predominantly early-phase studies and has produced mixed outcomes, ranging from acceptable feasibility in some regimens to limited efficacy, treatment-related toxicity, or no clear clinical benefit in others. Curcumin-containing combinations therefore require further validation through standardized synergy assessment, clinically achievable dosing strategies, comprehensive nanoformulation safety evaluation, pharmacokinetic–pharmacodynamic analysis, biomarker-guided patient selection, and well-designed prospective clinical trials. Overall, curcumin’s most credible future in oncology lies as a precisely delivered adjunct within rationally designed combination therapies supported by nanotechnology and guided by tumor biology.

## Data Availability

Data are contained within the article.

## Abbreviations

The following abbreviations are used in this manuscript:

CSC	Cancer stem cells
TME	Tumor microenvironment
ROS	Reactive oxygen species
NF-κB	Nuclear factor kappa B
PI3K/PKB	Phosphoinositide 3-kinase/protein kinase B
STAT3	Signal transducer and activator of transcription 3
MAPK	Mitogen-activated protein kinase
MDR	Multidrug resistance
EMT	Epithelial–mesenchymal transition
EGFR	Epidermal growth factor receptor
IGF-1R	Insulin-like growth factor 1 receptor
RNS	Reactive nitrogen species
IFN	Interferon
TGF-β	Transforming growth factor beta
EGR1	Early growth response 1
EGCG	Epigallocatechin-3-gallate
SLN	Solid lipid nanoparticle
5-FU	5-Fluorouracil
Cur	Curcumin
PTX	Paclitaxel
ER	Endoplasmic reticulum
NK cells	Natural killer cells
JNK	c-Jun N-terminal kinase
ERK	Extracellular signal-regulated kinase
HA	Hyaluronic acid
PEG	Polyethylene glycol
TNBC	Triple-negative breast cancer
AI	Artificial intelligence
